# Construction of a copper stepwedge with aluminum equivalent mean gray values

**DOI:** 10.1590/S1678-77572010000400012

**Published:** 2010

**Authors:** Nesrin DÜNDAR, Pelin GÜNERİ, Erinç ÖNEM, Hayal BOYACIOĞLU

**Affiliations:** 1 DDS, PhD, Ege University, Faculty of Dentistry, Department of Oral Diagnosis and Oral Radiology, Bornova, İzmir, Turkey.; 2 PhD, Ege University, Faculty of Science, Department of Statistics, Bornova, İzmir, Turkey.

**Keywords:** Aluminum, Copper, Radiography

## Abstract

**Objectives:**

To produce a copper (Cu) stepwedge with aluminum (Al) equivalent mean gray values (MGV).

**Material and Methods:**

The thicknesses of Cu steps that were equivalent to those of the Al were formulated using the X-ray attenuation properties of the materials. The Al and fabricated Cu stepwedges were radiographed, but the MGVs of the Cu stepwedge were mismatching to those of the Al. Using a mathematical function to adjust the pixel MGV of Cu stepwedge to those of the Al, new Cu stepwedges were created. *In vitro* iterations were performed until best approximation to Al was reached.

**Results:**

The MGV of the Cu stepwedges fabricated by formularization were different than those of Al (p=0.001). Iteration method led to MGV similar to those of the Al stepwedge (p=0.207).

**Conclusions:**

Construction of a Cu stepwedge according to the basic rules of radiophysic failed to result in a stepwedge with similar radiodensity values to those of Al stepwedge. Further studies may use the formularization method only for prototype Cu wedge production, but consecutive iterations shall be compassed to obtain the best approximation to Al MGV.

## INTRODUCTION

Optical density is defined as the logarithmic measure of the ratio of transmitted light to the incident light through the film image. It depends on the inherent X-ray attenuation properties of the materials and secondary parameters such as the film characteristics and exposure-processing conditions^16^. The lack of complete standardization of secondary parameters prevents the comparison of various radiographic data by using only quantified optical densities. Application of metal stepwedges that are radiographed alongside the objects is the general method to produce calibration curves to transpose the measured optical densities into equivalent thicknesses of the metal standards and to evaluate the hard tissue density^3,6,12,16^.

Aluminum (Al) has similar radiographic density to those of the bone and dentin, and it is thus the most common metal standard material that has been used in radiology for radiodensitometric assays^10^. It was established back in 1960 that radiographic contrast of 1 mm of mineralized tissue was equivalent to 1 mm of Al 1100^6^. In addition to Al, copper (Cu), nickel (Ni), cesium chloride (CsCl), calcium chloride (CaCl), ethanol and water have been used to compensate for the variations during film exposure, processing, and digitizing^7,11^. Among these materials, Cu stepwedge has been used occasionally^1,5,12^.It is a basic rule of radiophysics that in objects of the same thickness, the X-ray absorption of the material increases with the number of its electrons per centimeter^2,8^. The atomic number of Cu is 29, and its atomic density (atoms/cm^3^) is 8.45 x 10^22^, whereas these are 13 and 6 x 10^22^ for Al, respectively^8^. Therefore, Cu has higher x-ray attenuation when compared to Al. This characteristic is important for production of a thin stepwedge to use in conventional panoramic radiography machines, which are still in use in many clinics. In these machines, the distance between the film-cassette and the film-cassette holder machines is so narrow that it prevents the implantation of a standard Al stepwedge whose thickest step is 10 mm. Due to this technical limitation, the use of an Al metal standard in conventional panoramic radiography machines is impractical, and a thinner stepwedge is required for these machines. Because of its higher x-ray attenuation property, using Cu instead of Al would decrease the thickness of the stepwedge and would enable the application of metal calibration stepwedge in panoramic machines.

Additionally, it has been reported that aluminum is not a suitable metal standard for x-ray machines that contain Al filtration because the total effect of any Al would be reduced due to the filtration of the x-ray machine^13^. Consequently, metals other than Al would be more appropriate to employ stepwedges in these machines^13^. Construction of a Cu stepwedge whose steps’ radiodensity values are similar to those of the Al stepwedge would prevent this complication.

In studies that used Cu stepwedge instead of Al stepwedge, bone density has been expressed as "copper equivalent units"^1,12,13^. However, it would be more appropriate to express the actual bone density in terms of Al because it has radiographic density equal to the hard tissues^10^. This may be especially important when the variations need to be measured quantitatively on the conventional panoramic radiographs in order to determine the hard tissue density changes.

This study aimed to produce a Cu stepwedge with similar MGV to those of Al stepwedge that could be employed in conventional panoramic machines in order to offer a radiographic standardization device to be used for radiographic hard tissue density evaluations.

## MATERIAL AND METHODS

Because of the unique similarity of Al radiodensity to that of the bone, the Cu stepwedge also had to have the closest approximation to the radiodensity of Al. So, at the Department of Nuclear Physics, Faculty of Science, ege University, the x-ray attenuation properties of Cu and Al were employed in the following formula to determine the thicknesses of Cu steps equivalent to those of the Al:

*l*_X_=*l*_0_e^-(µ/ρ) ρX^,

where I_X_ is the intensity of transmitted beam after passing through a thickness *X*, I_0_ is the intensity of incident x-ray beam, µ is the linear absorption coefficient and ρ is density^2^. The values of mass absorption coefficient µ/ρ have already been established for various characteristic wavelengths used in diffraction, and it is 5.511 x 10^-2^ cm^2^/g for Al and 5.581 x 10^-1^ cm^2^/g for Cu in 80 kev^4^.

Therefore; if *l_X_*_Al_=*l*_0_e-(^µ/ρ.^ Al ^ρ^ Al ^*X*^ Al equals to *l_X_*_Cu_=l_0_e-(^µ/ρ^) Cu ^ρ^ Cu ^*X*^ Cu, then;

5.511 x 10^-2^ cm^2^/g x 2.699 g/cm^3^ x 0.1 cm = 5.581 x 10^-1^ cm^2^/g x 8.969 g/cm^3^ x Cu

The results presented in Table 1 were obtained.

**Tabela 1:** Thicknesses of Cu steps equivalent to those of Al, established according to the x-ray attenuation coefficient properties of the materials

**Al (mm)**	**Cu (mm)**
	
1	2.974495 x10-3cm x10 = ~0.03
2	5.948990 x10-3cm x10 = ~0.06
3	8.923485 x10-3cm x10 = ~0.09
4	1.189798 x10-2cm x10 = ~0.12
5	1.487248 x10-2cm x10 = ~0.15
6	1.784697 x10-2cm x10 = ~0.18
7	2.082146 x10-2cm x10 = ~0.21
8	2.379596 x10-2cm x10 = ~0.24

**Figure 1:**
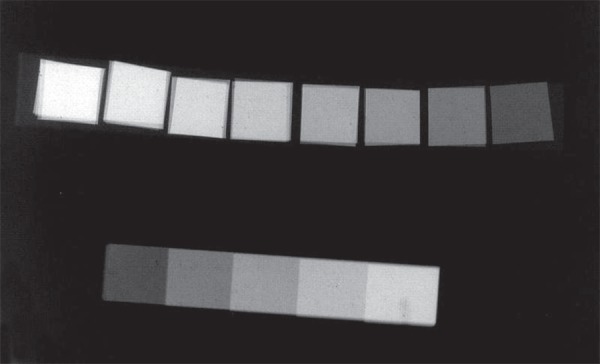
Radiograph of the prototype Cu stepwedge formed according to the x-ray attenuation properties and with 0.03-0.24 mm step thicknesses. A standard Al stepwedge with 5 incremental steps ranging between 2-10 mm is also shown

A 0.03-mm-thick copper sheet was used to produce the copper stepwedge. The purity of copper and aluminum was tested by using inductive coupled plasma optic emission spectroscopy (ICP/OeS) (Parkin/elmar Optima 2100, DV) after digestion of copper with nitric acid and aluminum with hydrochloric acid. The results showed that the purity of aluminum was 94.7% and copper was 99.99%. Then, a prototype of Cu stepwedge that had 8 incremental steps ranging between 0.03-0.24 mm was produced by folding the copper sheet. This prototype wedge and a standard Al stepwedge, which was machined and had 5 incremental steps ranging between 2-10 mm, were placed on a size 2 periapical E speed film (Ceadent, Strängnäs, Sweden). The dose-response curve of the film was controlled. Under standard conditions (70 kVp, 10 mA, 0.4 s), the wedges were radiographed using a standard radiography device (Trophy Radiologie, 77437, Croissy-Beabourg, France) (Figure 1) with inherent 2-mm Al filtration. The radiographs were processed in an automatic processing machine (Dürr XR-04, Dürr Dental, Bietigheim, Germany) and were digitized by a scanner with a transparent adapter (epson eXP 1680Pro, Seiko epson Corp., Nagano, Japan). This procedure was repeated on three occasions and digital images of 3 different radiographs were obtained.

The mean gray values (MGV) of each step of Cu and Al stepwedges were measured by using the histogram function of a computer graphics program (Adobe Photoshop 8.0, Adobe Systems Inc, San Jose, CA, USA). For each step of each stepwedge, the center was marked as 50 x 50 pixel region of interest (ROI). Then, 3 measurements were performed and the mean MGV of that step was calculated (Table 2).

**Tabela 2:** Mean MGV values and standard deviations of each step of Al and prototype Cu stepwedges

**Al steps**	**Mean gray values and sandard deviation **
	
1	96.193±5.22
2	130.797±5.82
3	158.543±7.28
4	83.867±7.81
5	205.400±7.56
	
**Cu steps**	**Mean gray values and sandard deviation**
	
1	105.037±5.26
2	132.117±5.51
3	153.707±5.96
4	168.673±6.31
5	182.773±6.73
6	194.820±6.24
7	202.327±6.03
8	209.637±6.11

**Figure 2:**
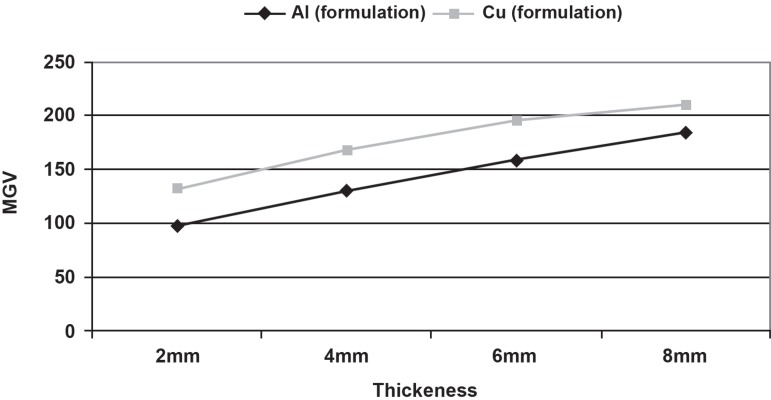
Mean MGVs of the Al and prototype Cu stepwedges formed according to the x-ray attenuation properties, revealing the quadratic sensitometric curves of the stepwedges

When the distribution of the data was analyzed accordingly, it was observed that the sensitometric curves of both stepwedges were quadratic. The measured MGV of the Cu stepwedge which was produced by using a basic rule of radiophysics that was related to the x-ray attenuation properties of Cu and Al were not even close to the MGV of the Al stepwedge (Figure 2). This result showed that the formularized approach was not successful in clinical conditions. Therefore, a mathematical function that automatically adjusted the pixel MGV of Cu stepwedge to those of the Al stepwedge was developed. The required thicknesses of the steps of Cu stepwedge were calculated using the following equation:

ŷ= 23.79 + 955.657x - 1005.5x^2^,

and the new results were obtained (Table 3).

**Tabela 3:** Required thicknesses of the Cu stepwedge steps, calculated according to the acquired equation

**Steps**	**Thickness of Cu (mm)**	**Mean MGVs (adopted from those of the AI)**
		
1	0.027	49.58
2	0.07	85.75
3	0.115	120.40
4	0.15	144.54
5	0.19	169.06

However, the 1st and 3rd steps of the Cu wedge were too thin to be fabricated in our laboratory facilities. Therefore, the plausible thicknesses that were closest to the original values were calculated (Table 4).

**Tabela 4:** Plausible Cu thicknesses that were closest to the original values

**Steps**	**Thickness of Cu (mm)**	**Mean MGVs (adopted from those of the AI)**
		
1	0.03	51.59
2	0.07	85.75
3	0.11	116.74
4	0.15	144.54
5	0.19	169.06

**Figure 3:**
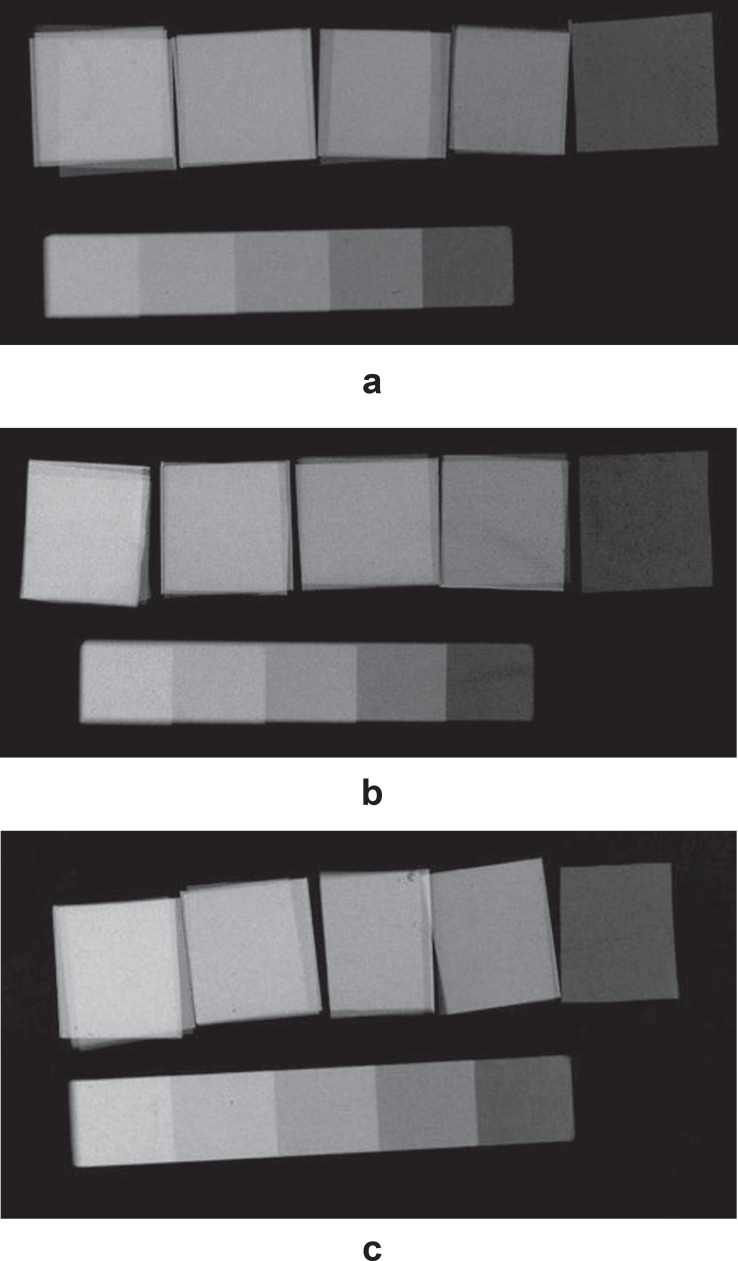
Three Cu stepwedges were fabricated and were radiographed with Al stepwedge in order to measure the agreement of the radiopacities of Cu thicknesses with those of the aluminum. (Step thicknesses of Cu=0.03, 0.07, 0.11, 0.15, 0.19 mm; Al=2, 4, 6, 8, 10 mm) (a: Cu stepwedge 1, b: Cu stepwedge 2, c: Cu stepwedge 3)

Three new Cu stepwedges were formed according to these renewed step thicknesses. The accuracy of these stepwedges was tested in order to see whether they could provide the expected MGV approximations to those of the Al stepwedge. Cu stepwedges that were accompanied by standard 5 steps Al stepwedge were radiographed, processed and digitized separately, as mentioned before (Figures 3a-c). The MGV of each step of the stepwedges were measured 3 times, and the mean MGV value of each step was established. This procedure was sustained until a Cu stepwedge that had similar MGV to those of the Al stepwedge was produced. These trials were called as "iteration" because new thickness calculations, x-ray exposures and MGV measurements had to be repeated until the best approximation to those of Al was reached. When the thicknesses of the Cu stepwedge steps were revised as follows, the results of Cu stepwedges provided best approximation to those of the Al stepwedges (Table 5).

**Table 5:** The thickness of Cu stepwedges provided by best approximation to those of the Al stepwedges

**Steps**	**Thickness of Cu (mm)**
	
1	0.03
2	0.06
3	0.10
4	0.14
5	0.18

The MGV data of the Al and Cu stepwedge that was produced by formularization were analyzed by paired t tests to determine the significance of the differences between the stepwedges. The same statistical analyses were repeated for the MGV data of the Al and Cu stepwedge that was developed according to the iteration method. In all tests, alpha was set as 0.05.

## RESULTS

In addition to the thicknesses of the materials, the purity and the exposure-processing parameters are the other major components of radiographic density. Therefore, it shall be noted that the measurements presented hereby would be valid only for this study and the above-mentioned factors may change the results in other test conditions.

In all tests, Al stepwedge was accepted as the "gold standard". The validity of both methods (formularization and iteration) that were employed to produce a Cu stepwedge was determined by investigating the approximation of the MGV of the Cu stepwedges to those of the Al stepwedge.

**Figure 4:**
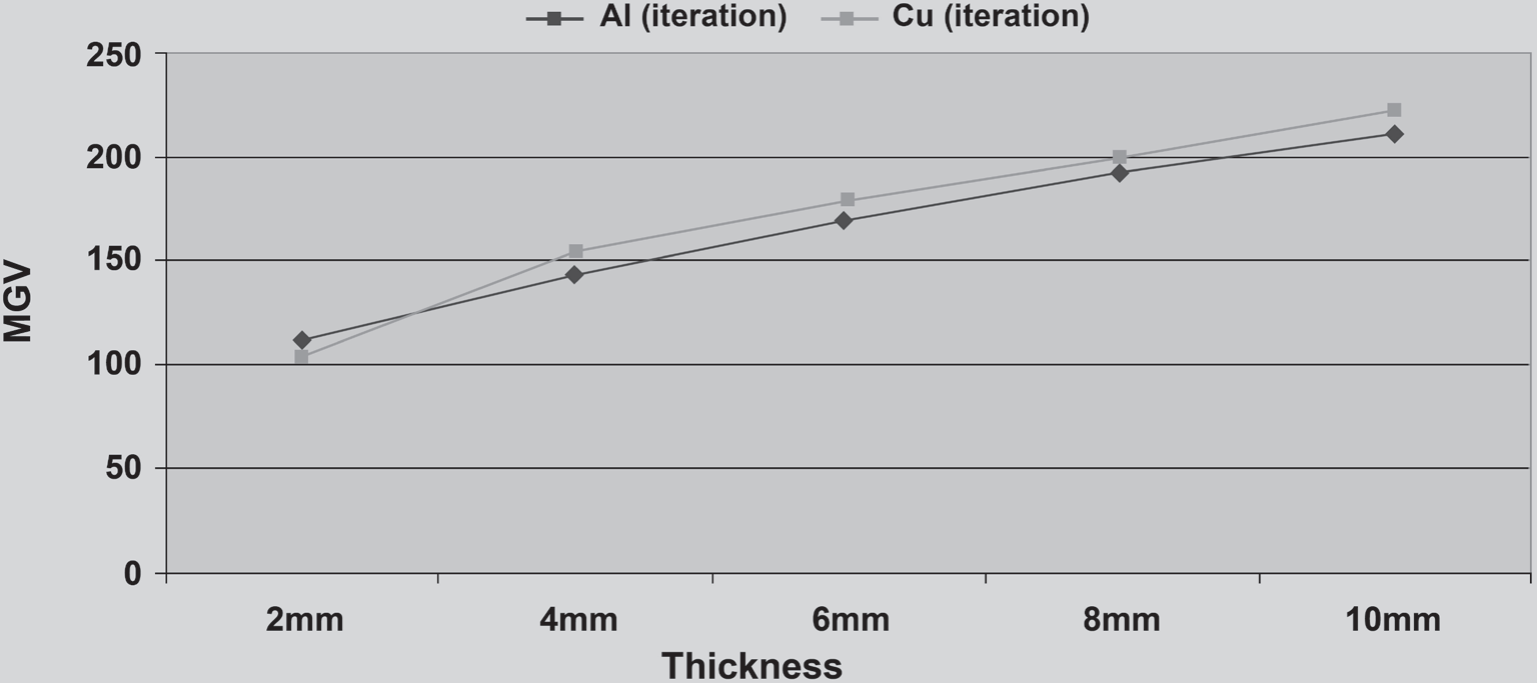
Sensitometric curves of the stepwedges obtained by the MGVs of (A) the reciprocating steps of Al and Cu stepwedges designed according to the formulation method using the x-ray attenuation properties (MGVs p=0.001); and (B) of Al and Cu stepwedges, when Cu stepwedge was produced with iterations (MGVs p=0.207)

The MGV values and standard deviations of each step of Al stepwedge and the prototype Cu stepwedges are presented in Table 2. Those values obtained with Al and revised Cu stepwedges are shown in Table 6. Comparison of the MGV of the Al stepwedge and Cu stepwedge which was produced by using the formularization method (employing the x-ray attenuation properties of the materials) is shown in Figure 4a. The same comparison was done with the Cu wedge, which was produced with iteration method, and the results are presented in Figure 4b.

**Table 6:** MGVs and standard deviations of three Al stepwedges with 2-10-mm-thick steps, and three Cu stepwedges with 0.03, 0.06, 0.10, 0.14, and 0.18-mm-thick steps

**Al steps**	**Mean MGV1**	**Mean MGV2**	**Mean MGV3**	**Total Mean**
				
1	64.120±3.42	78.410±5.07	85.383±3.99	75.971
2	97.513±4.29	124.160±5.56	127.323±4.04	116.332
3	124.773±4.25	160.500±5.59	163.687±4.34	149.653
4	144.647±4.55	184.557±6.12	191.067±5.12	173.423
5	161.543±4.70	204.867±6.72	212.543±4.82	192.984
				
**Cu steps**	**MGV1**	**MGV2**	**MGV3**	**Mean**
				
1	59.603±3.57	73.720±5.24	78.103±3.64	70.476
2	106.863±4.33	140.707±6.25	144.960±4.69	130.843
3	129.473±4.27	167.640±6.15	170.323±4.71	155.812
4	146.213±4.23	189.127±6.24	193.423±4.75	176.254
5	164.783±5.08	214.647±7.21	218.263±4.62	199.231

As observed, the MGV of the Cu stepwedge that was produced by the formulation method were significantly different (p=0.001) from those of the Al stepwedge. However, the MGV of the Cu stepwedge that was designed using the iterations method were statistically similar (p=0.207) to the MGV of the Al stepwedge.

## DISCUSSION

The radiodensity of the hard tissues is an applicative tool for determination of the bone changes, either as a bone loss (radiolucency) or gain (radiopacity). Radiopacity is largely governed by the purity of the material and the characteristics of the incident x-rays. The properties of x-rays are influenced by the target material, the energy distribution and the number of incident photons^14^. Additionally, photons emitted at different angles pass through different thicknesses of the material and the attenuation characteristics within an x-ray beam vary with the off-axis distance^9^. So, the interaction between photons and atoms involves complex physical processes which effect the x-ray attenuation measurements, such as the scattering of the incident photons by the absorbing material, and the influence of the air path and the x-ray fluorescence^3,15^. In order to provide exactly the same MGV results in each radiographic test session, the spectrum and intensity of the incident x-ray, and the interaction between the incident photons and the matter shall be perfectly replicated in each experiment. However, as observed in our study, this is not achievable in clinical practice^3,15^. The present results also showed this phenomenon and successive iterations had to be performed until a valid "thickness determination" was reached.

McArthur and Taylor^13^ (1975) have found that 0.08 mm Cu and 2.10 mm Al produced equal optical radiopacities. Those authors stated that the Cu shall be at least 0.035 mm thick for 50% observability, whereas it shall have a minimum of 0.077 mm thickness for 95% observability^13^. Our digital analysis method using MGV measurements revealed that the radiodensity of 0.03 mm Cu was equivalent to 2 mm Al, and the thicknesses of the steps of the proper Cu stepwedge ranged between 0.03 and 0.18 mm. These values were close to those reported by McArthur and Taylor^13^ (1975), that is, 0.035 to 0.077 mm. The discrepancies of the results between these two studies may be explained by employment of different techniques (optical density and MGV measurements).

In the literature, when the actual numerical value of radiopacity is required, a simple algebraic calculation which is based upon linear regression against thickness shall better be performed, instead of relying upon solely graphical interpolation of optical density versus thickness^16^. This has been developed by application of the following equation:

log (*d_i_*)= (*C*-OD_*i*_)/-*m,*

where OD= optical density, i= any material on the x-ray film, *m* = gradient or slope of the curve of optical density versus log_10 _step height, *C* = the interception on the OD axis, *d* = step height^16^. Nevertheless, the slopes of the regression lines may show slight differences because of the material micro-density variations, which lead to small but important variation of radiopacity within and between successive specimens of the same geometry (radiographic inhomogenity)^16^.

What was tested in the present investigation may seem as a routine procedure that has been in use for decades. However, the mismatch of the radiodensity values of Cu and Al stepwedges observed in the initial part of this study guided us to the construction of a new Cu stepwedge by application of MGV of the materials. A mathematical approach was utilized to determine the thickness of each step of a Cu stepwedge by transcribing the pixel MGV of Al stepwedge. Therefore, a Cu stepwedge whose each step has similar MGV to those of Al was formed. Using this method, 3 new Cu stepwedges were produced and were subject to subsequent radiodensitometric analyses in order to test the reproducibility. During these analyses, material micro-density variations, which may lead to radiopacity variations^16^ due to the beam hardening effect, were also observed in this study and prevented the exact matching of the MGV of Cu and Al. However, the final Cu stepwedge that had steps ranging between 0.03-0.18 mm produced MGV similar to those of the Al, and showed the same analogy in each consecutive test. Considering that the formularization method has been widely used in the literature and our approach has not been applied previously, the validity of those Cu stepwedges as Al equivalent calibration standards may be questionable.

The advantage of this stepwedge may be the elimination of any further conversion of Cu values into Al measurements to obtain "hard tissue equivalent" results. So, radiographic density of any point on the radiograph would be expressed as "Al equivalent". even though digital panoramic radiography machines are gaining increasing popularity in head and neck imaging, there are many clinics in developing countries that still use conventional panoramic radiography machines. The present method may be especially helpful when hard tissue variations, such as bone healing or degeneration rate, as well as the treatment outcomes concerning the hard tissues need to be measured on conventional panoramic radiographs,. However, it must be underlined once again that this Cu stepwedge was formed under the tested exposure/ processing conditions by using 99.9% pure Cu and 94.7% pure Al. Therefore, it is suggested that, in future experiments, the formularization method is employed only for the production of a prototype wedge. Then, consecutive multiple iterations shall be compassed to reach a better approximation to the radiodensity of Al. Additionally, the validity of the presented thicknesses of copper stepwedge shall be further tested with successive *in vitro* and *in vivo* tests using both healthy and osteoporotic bone specimens.

## CONCLUSION

The construction of a Cu stepwedge according to the basic radiophysics rules failed to result in a stepwedge with similar radiodensity values to those of Al stepwedge. Further research may use the formularization method only for prototype Cu wedge production, but consecutive iterations shall be compassed to obtain the best approximation to Al MGV.
